# Supplementary figures and images for: Platelet count/spleen volume ratio has a good predictive value for esophageal varices in patients with hepatitis B liver cirrhosis
Source: PLoS One. 2021 Dec 2;16(12):e0260774. doi: 10.1371/journal.pone.0260774 (PMC8638864; doi:10.1371/journal.pone.0260774)

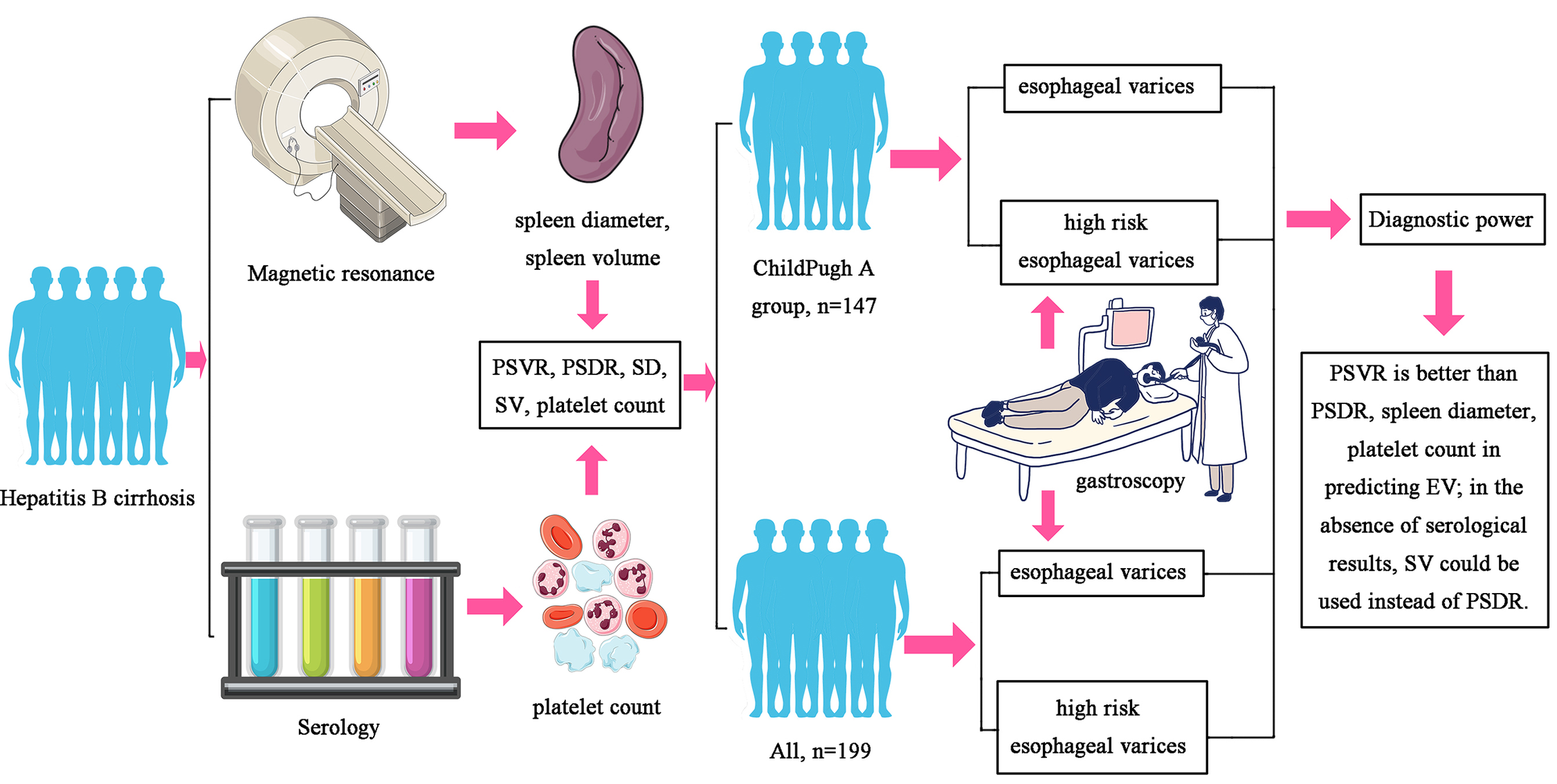

Supplement: S1 Graphical abstract — (TIF) [file pone.0260774.s003.tif]
