# Supplementary material for: Platelet count/spleen volume ratio has a good predictive value for esophageal varices in patients with hepatitis B liver cirrhosis
Source: PLoS One. 2021 Dec 2;16(12):e0260774. doi: 10.1371/journal.pone.0260774 (PMC8638864; doi:10.1371/journal.pone.0260774)
Supplement: Attachment — Submitted filename: Rebuttal letter.docx [file pone.0260774.s005.docx]

Xiangyang No.1 People's Hospital, November 10, 2021

Responses to Academic editor and Reviewers

**Professor Gopal Krishna Dhali**

**PLOS ONE**

**Title:** Platelet count/spleen volume ratio has a good predictive value for esophageal varices in patients with hepatitis B liver cirrhosis

**Authors:** Sihao Yu, Wei Chen, Zicheng Jiang

**Manuscript ID:** PONE-D-21-33951

Dear Editors and Reviewers:

Thank you for your letter dated November 10. We were pleased to know that our work was rated as potentially acceptable for publication in Journal, subject to minor revision. We thank the editors and reviewers for the time and effort that they have put into reviewing the previous version of the manuscript. Their suggestions have enabled us to improve our work. Based on the instructions provided in your letter, we uploaded the file of the revised manuscript. Accordingly, we have uploaded a copy of the original manuscript with all the changes highlighted in MS Word. Appended to this letter is our point-by-point response to the comments raised by the reviewers. The comments are reproduced and our responses are given directly afterward in a different color (red). We would like also to thank you for allowing us to resubmit a revised copy of the manuscript.

We hope that the revised manuscript is accepted for publication in the Journal of PLOS ONE.

The modified parts are marked in red in the copy paper. The main corrections in the paper and the responds to the academic editor and reviewers’ comments are as following:

Responds to the academic editor’s comments:

Since this is a retrospective study and the methods are written in detail in the Materials and methods and Statistical analysis sections, there is no need to store it in protocols.io.

1. Please ensure that your manuscript meets PLOS ONE's style requirements, including those for file naming. The PLOS ONE style templates can be found at
<https://journals.plos.org/plosone/s/file?id=wjVg/PLOSOne_formatting_sample_main_body.pdf> and <https://journals.plos.org/plosone/s/file?id=ba62/PLOSOne_formatting_sample_title_authors_affiliations.pdf>

We have carefully read the documents PLOSOne_formatting_sample_main_body.pdf and PLOSOne_formatting_sample_title_authors_affiliations.pdf for PLOS ONE's style requirements, file naming requirements and made changes accordingly.

2. Please review your reference list to ensure that it is complete and correct. If you have cited papers that have been retracted, please include the rationale for doing so in the manuscript text, or remove these references and replace them with relevant current references. Any changes to the reference list should be mentioned in the rebuttal letter that accompanies your revised manuscript. If you need to cite a retracted article, indicate the article’s retracted status in the References list and also include a citation and full reference for the retraction notice.

We reviewed the references again to ensure that they were complete and correct.

Additional Editor Comments:
Kindly correct the numerical error on page 9, line 166 and page 12, line 229.

We have corrected 57% to 67% (page 9, line 162 and page 21, line 276). We are very sorry for the trouble we have caused you.

Thank you very much for your dedication!

Responds to the reviewer’s comments:

Reviewer #1: Well conceptualized study. Thanks to the authors for picking up this particular issue for their investigation. Statistical analyses were performed adequately as was suggested. Manuscript contains all the data related to the study observations. Authors complied with the suggestions provided in the previous review of the manuscript (ID: PONE-D-21-30287). I would request authors to continue this study with the compensated cirrhosis only.

Response to comment: Thank you for your compliments and we will continue to look for more meaningful inspiration. We will consider the next study in a population with compensated cirrhosis. Thank you very much again for your advice!

Reviewer #2: 1. Well thought concept.

Response to comment: Thank you for your praise and we will continue to work on more creative attempts.
2. English grammar can be improved for the entire article.

Response to comment: Our manuscript has been corrected in English grammar by 3 people since the beginning, but it still doesn't work very well, if possible, can you tell us where the problem is very big? Thank you very much for your advice.
3. Following correction to be made.
(See line 158, page 9): Mean age and range/IQR is reported. It should be median and range/IQR.

Response to comment: We are very sorry for our repeated mistakes and have corrected the phrase. (See line 154, page 9)

Thank you very much for your comments, we will work harder to learn English to make the language more vivid.

We sincerely thank the editors and reviewers for their enthusiastic work and hope that the corrections will be accepted.

Again, thank you so much for your comments and suggestions!

Best regards

Sincerely yours

Sihao Yu

(On behalf of co-authors)

Detailed address

Tel: (+86)15997812001	Email: [1034137401@qq.com](mailto:1034137401@qq.com)
